# Stem Cell Conditioned Medium Treatment for Canine Spinal Cord Injury: Pilot Feasibility Study

**DOI:** 10.3390/ijms21145129

**Published:** 2020-07-20

**Authors:** Zuzana Vikartovska, Maria Kuricova, Jana Farbakova, Tomas Liptak, Dagmar Mudronova, Filip Humenik, Aladar Madari, Marcela Maloveska, Eva Sykova, Dasa Cizkova

**Affiliations:** 1 Center of Experimental and Clinical Regenerative Medicine, University of Veterinary Medicine and Pharmacy, Komenskeho 73, 04181 Kosice, Slovakia; zuzana.vikartovska@uvlf.sk (Z.V.); filip.humenik@uvlf.sk (F.H.); marcela.maloveska@uvlf.sk (M.M.); 2 University Veterinary Hospital, University of Veterinary Medicine and Pharmacy, Komenskeho 73, 04181 Kosice, Slovakia; maria.kuricova@uvlf.sk (M.K.); jana.farbakova@uvlf.sk (J.F.); tomas.liptak@uvlf.sk (T.L.); aladar.madari@uvlf.sk (A.M.); 3 Department of Microbiology and Immunology, Institute of Immunology, University of Veterinary Medicine and Pharmacy, Komenskeho 73, 04181 Kosice, Slovakia; dagmar.mudronova@uvlf.sk; 4 Institute of Neuroimmunology, Slovak Academy of Sciences, Dubravska cesta 9, 845 10 Bratislava, Slovakia; sykovae@gmail.com

**Keywords:** spinal cord injury, canine, mesenchymal stem cells, conditioned medium, regenerative medicine

## Abstract

Spinal cord injury (SCI) involves nerve damage and often leads to motor, sensory and autonomic dysfunctions. In the present study, we have designed a clinical protocol to assess the feasibility of systemic delivery of allogenic canine bone marrow tissue-derived mesenchymal stem cell conditioned medium (BMMSC CM) to dogs with SCI. Four client-owned dogs with chronic SCI lasting more than six months underwent neurological and clinical evaluation, MRI imaging and blood tests before being enrolled in this study. All dogs received four intravenous infusions with canine allogenic BMMSC CM within one month. Between the infusions the dogs received comprehensive physiotherapy, which continued for three additional months. No adverse effects or complications were observed during the one, three and six months follow-up periods. Neither blood chemistry panel nor hematology profile showed any significant changes. All dogs were clinically improved as assessed using Olby locomotor scales after one, three and six months of BMMSC CM treatment. Furthermore, goniometric measurements revealed partial improvement in the range of joint motion. Bladder function improved in two disabled dogs. We conclude that multiple delivery of allogenic cell-derived conditioned medium to dogs with chronic SCI is feasible, and it might be clinically beneficial in combination with physiotherapy.

## 1. Introduction

Spinal cord injury (SCI) in dogs, similarly as in humans, leads to devastating conditions, including paresis or paralysis associated with urinary and fecal incontinence [1,2,3]. Injury to the spine is commonly documented in breeds susceptible to prolapsed-herniated intervertebral discs (IVDD), such as dachshund, beagle, poodle or bulldog [4,5]. Traumatic exogenous insults such as falls or motor vehicle accidents may also lead to SCI. Prognosis for functional recovery depends on the initial severity (incomplete/complete lesion), location of the injury (cervical, thoracic, lumbar) and general health and age of dogs [6]. Care during the first two to four days (decompression, stabilization of vertebral column) followed by therapy and rehabilitation are critical for initiating the recovery outcome [7]. While some SCI dogs receiving the appropriate medical care improve their quality of life and motor function, usually within six months, others proceed into the chronic phase. The management of secondary complications during the chronic phase is more difficult and often does not lead to gradual improvement [8].

Due to the complex anatomy of the spine and its limited regenerative capacity in mammals, there is currently no cure for SCI [9]. Partial successes have been achieved in acute SCI by enrolling combined treatment strategies and targeted rehabilitation [6,7]. Many traditional therapeutic approaches stabilize or alleviate symptoms. The use of methylprednisolone, known as the gold standard for the treatment of acute SCI in humans as well as in dogs for many years, is nowadays highly controversial [10,11]. There are therefore, still major challenges in designing advantageous therapies for such a complex scenario as SCI [12]. Experimental approaches using different types of stem cells or even their conditioned medium are undergoing extensive research [13,14,15,16]. Adult mesenchymal stem cell-based therapy in general modulates oxidative stress, and secreted cytokines and growth factors have immunomodulatory, anti-inflammatory, pro-angiogenic and anti-apoptotic effects [17]. Although promising results from experimental stem cell research (rats, mice) have been achieved, most of these strategies do not translate into clinical practice due to loss of efficiency in human trials [16]. Thus, there is a need to test these strategies on larger animals which more closely resemble the anatomy and immune response in humans, and which allow us a sufficiently long follow-up observation period. 

Dogs with SCI are nowadays recognized as an important translational model for filling the gap between experimental rodent models and clinical practice [16]. Mesenchymal stem cells (MSCs) generated from adult and neonatal tissues have been enrolled for experimental treatment of canine SCI [18,19]. In addition, extracellular vesicles have emerged as an important bioactive component of the MSC conditioned media which may be useful for intervention treatment [20]. Olfactory ensheathing cells (OECs) revealing persistent neurogenesis seem to be another good candidate for treatment of canine experimental and naturally-occurring SCI [20,21]. Dogs treated with OECs showed better coordination and increased pelvic limb movement when compared with controls [22,23]. Unlike Schwann cells, which are also able to regenerate damaged nerve tissue, OECs have better interaction with astrocytes and lead to reduced glial scar formation [24].

However, identifying the optimal cell type candidate with the highest neuroprotective effect is the key condition for successful therapy. According to our genomic, proteomic and functional study, canine bone marrow-derived MSCs seem to be one of the best candidates, since they release a variety of bioactive molecules possessing strong paracrine factors which may affect various neurological disorders [25].

Based on our knowledge from in vitro MSC profiling [17] and in vivo SCI treatment strategies [16], we have designed a pilot study involving clients’ dogs with naturally-occurring SCI. The main goal of our study was to design and optimize the treatment protocol for canine bone marrow-derived mesenchymal stem cell conditioned medium (BMMSC CM) delivery and rehabilitation in dogs suffering from chronic SCI. In addition, we assessed the feasibility and safety of multiple BMMSC CM systemic delivery in the veterinary clinical setting for at least six months.

## 2. Results

### 2.1. Flow Cytometry with Canine CD Markers

**Figure 1:**
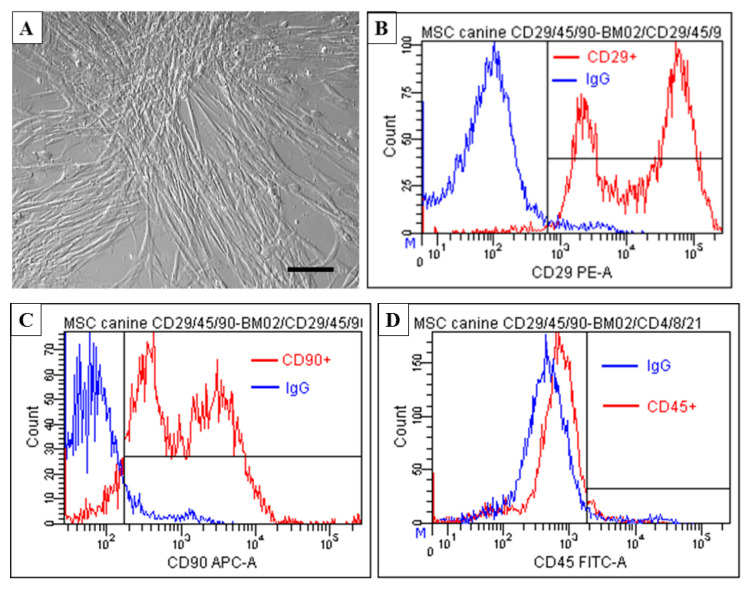
Immunophenotyping of bone marrow tissue-derived MSC (BMMSC) at passage 3. (**A**) Note confluent population of BMMSC revealing spindle-shaped morphology. (**B**–**D**) Flow cytometry analyses of BMMSC expressing high percentage of mesenchymal markers CD29+ (88.5 ± 2.2%) and CD 90+ (77.3 ± 4.6%) and low percentage of hematopoietic markers CD 45+ (3.0 ± 1.7%). Scale bar A = 50 μm.

Flow cytometry analyses confirmed that the population of BMMSC expresses a low number of surface markers typical for hematopoietic cells (CD45), while expression of surface markers typical for MSCs (CD29 and CD90) is high. The results presented in Figure 1 show that cells at passage 3 expressed primarily CD29 (88.5 ± 2.2%) and CD90 (77.3 ± 4.6%) and the proportion of CD45+ cells was low (3.0 ± 1.7%).

### 2.2. Multilineage Potential

**Figure 2:**
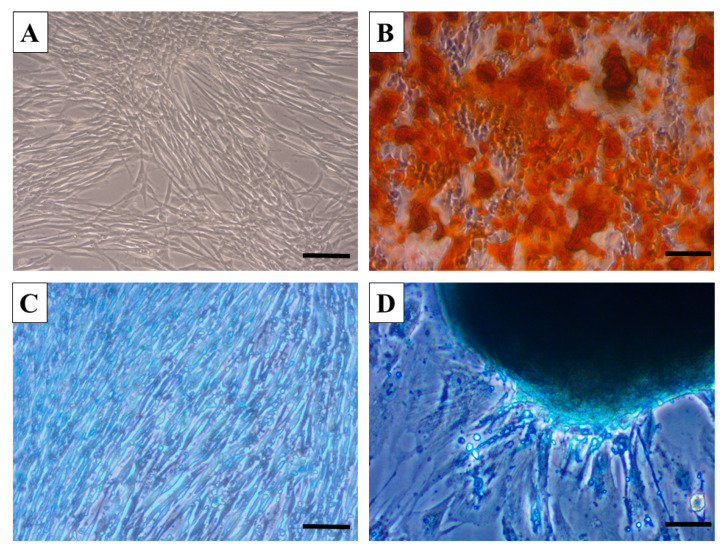
Micrographs documenting multipotent characteristics of canine BMMSC. Note differentiation into osteocytes (**B**, Alizarin Red) compared to control (**A**) and chondrocytes (**D**), Alcian Blue) compared to control (**C**). Scale bars A, B, C, D = 50 μm.

Three-lineage potential was confirmed using the commercial StemPro^®^ Differentiation Kit and corresponding protocol for induction towards chondrogenic, osteogenic and adipogenic lineages. After 14 days of incubation in cell-type corresponding differentiation medium, we confirmed that canine BMMSC show a high degree of biomineralizing osteogenesis with visual staining of calcium deposits expressing Alizarin Red positivity (Figure 2B). Furthermore, chondrocytes migrating from spherical chondrocyte-like aggregates revealed intense Alcian Blue staining of glycoproteoglycans (Figure 2D). In contrast to the high osteogenic and chondrogenic differentiation of canine BMMSC, we found a low degree of adipogenesis, with limited vacuole formation and Oil Red O staining (data not included). 

### 2.3. Locomotor and Bladder Function

**Figure 3:**
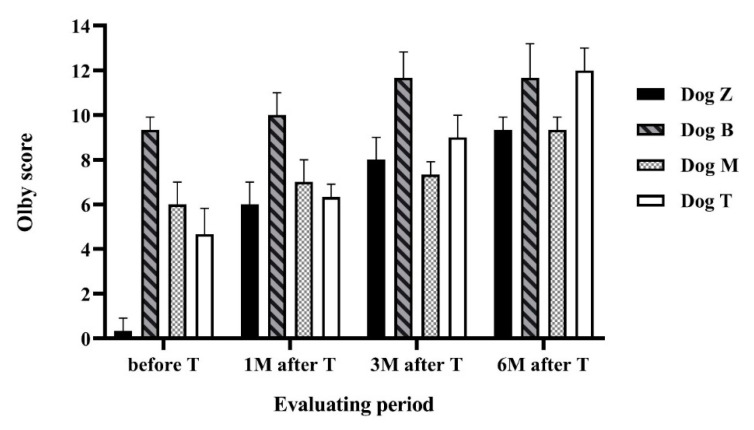
Locomotor function evaluated using Olby scale. The figure shows the Olby scores assessed in every dog during the study period. Values were obtained before therapy, then one, three and six months after therapy in each dog. Each measurement represents mean value of three measurements.

Locomotion scores in all dogs gradually increased with the treatment, as evaluated using the Olby locomotion scale (Figure 3). After the treatment and during the six months follow-up period all dogs improved in all evaluated parameters (locomotion scales, urinary bladder function, and goniometric measurements).

The gait ability and locomotion scores are expressed in Figure 3. Dog Z improved from 0 to 9. At the beginning of the study the dog was unable to walk at all, there was no pelvic limb movement and no deep pain sensation in the hind limbs. Six months after the treatment the dog was weight-bearing with reduced strength in the pelvic limbs. Failures such as crossing and scuffing the legs were visible more than 90% of the time. Dog M improved from 5 to 9, meaning from non-weight-bearing protraction of the pelvic limbs to weight-bearing protraction with more than 90% failures. Dog B improved from 9 to 13, which means improvement from more than 90% failures during walking to ataxic pelvic limb gait with normal strength and with failures such as incoordination and bunny-hopping visible less than 50% of the time. Dog T improved from 4 to 12, from non-weight-bearing protraction of the pelvic limbs with visible movement in more than one joint less than 50% of the time to ataxic gait with normal muscle strength and failures visible more than 50% of the time. 

In the two dogs with urinary retention, unable to spontaneously urinate, we reported mild improvement in urination ability. Both dogs had score 0 prior to the treatment. We used a urinary function score designed for the purposes of this study. Dog Z became easy to express and achieved the score 1. The Dog M gained score 2.

### 2.4. Goniometry Measurements

Dog Z showed improvement in almost all joints measured except flexion of the shoulder, stifle and extension of the hip (Table 1A). Dog B improved in all measured joints (Table 1B). Dog M demonstrated slight improvement in the pelvic limbs, in extension as well as flexion, with no improvement or a little worsening in the thoracic limbs (Table 1C).

### 2.5. Blood Analyses

**Figure 4:**
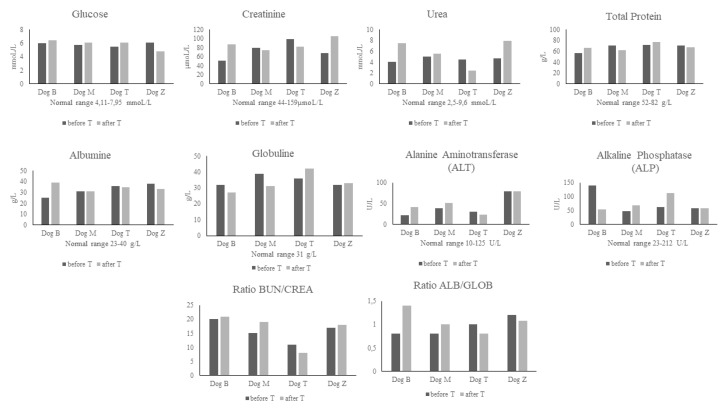
Biochemistry parameters before treatment (before T) and after treatment (after T), blood urea nitrogen (BUN), creatinine (CREA), ALB (albumin), GLOB (globulin).

Patients blood samples analyzed before treatment did not show any significant deviations from the normal range of chemistry and hematology parameters (Figure 4 and Table 2). This confirmed our assumption that all four dogs were healthy except for the motoric disruption associated with spinal injury. Blood chemistry results after treatment confirmed that our cell-free therapy was safe and did not have negative effects on liver and kidney, or other vital organ function. Similarly, the results from hematology did not reveal any significant changes from the normal range of parameters, except in two cases. Dog M (German Shepherd) had a slightly elevated mean platelet volume (MPV) level before treatment, while after treatment the MPV dropped to the normal physiological range. The second Dog T (Bichon) showed decreased mean cell volume (MCV) after treatment. However, while we have to state that the changes in each given parameter were slightly shifted from the normal range in both dogs, this was without any harmful clinical impact on the patients. 

## 3. Discussion

Chronic spinal cord injury is a serious problem which so far has no effective treatment. Although in recent years significant advances in stem cell technology, biomaterial and immunomodulation have been accomplished in experimental rodent models of SCI [26,27], their application in clinical contexts requires studies in higher mammals. In the present study we confirm the feasibility of a treatment protocol allowing optimal administration of BMMSC CM combined with comprehensive rehabilitation in dogs with SCI. No adverse effects or complications were observed in any of the treated dogs during the entire follow-up period. They showed locomotor improvement with enhanced range of joint motion, and in two disabled dogs more balanced bladder function was also achieved. 

Previous studies showed poor outcomes in dogs processed for experimental spinal cord injury without treatment. These dogs showed less locomotor improvement as assessed with Olby scores compared with dogs treated with MSCs [28] or neural stem cells [29].

Most treatments focus on acute SCI, where improvement is more likely. However, a large proportion of dogs progress to the chronic stage, where only symptomatology treatment with ongoing rehabilitation are available.

The majority of pet owners prefer non-invasive therapy, given by systemic administration, most often in the form of infusion solutions. Intravenous delivery of stem cell-derived conditioned media may therefore provide a clue to solving the challenges which current therapies cannot meet. This innovative cell-free strategy may act on many aspects of SCI secondary processes, avoiding the disadvantages and ethical concerns related to the use of stem cells. However, one of the most important factors directly affecting the success of treatment is the selection of well-defined stem cells for secretion of the conditioned medium.

For this reason, in the present study we decided to include canine BMMSC, which possess a typical mesenchymal immune profile and multilineage capacity. Furthermore, the standardized procedure for obtaining BMMSC and producing corresponding CM were well defined in our previous comprehensive genomic, proteomic and functional study, confirming their paracrine effect by releasing various pro-regenerative mediators, including chemokines, cytokines, growth and neuroprotective factors, as well as immune-modulatory proteins affecting adaptive and innate cells [25]. Although, they were differentiated into osteogenic and chondrogenic lineages, low adipogenesis occurred. Similar features have been detected in other studies [30]. This might presume a limitation of BMMSC multilineage potential. However, according to the StemPro Adipogenesis Differentiation Kit producer recommendation, we used, as a standard, 14 days of incubation, which was an appropriate time for osteogenic and chondrogenic, but revealed low adipogenic differentiation. Insufficient adipogenesis can be also a result of intensive osteogenesis. There is evidence that osteogenesis can inhibit adipogenesis and vice versa [31]. This process works through suppression of peroxisome proliferator-activated receptor γ (PPAR γ) serving as a transcription protein to adipocytes. This inhibition is secured by BMP and Wnt, which act as activators of osteogenic differentiation [31]. Osteogenic potential can be influenced by various factors, such as donor age or inflammation [32,33]. This may be an explanation for the poor adipogenesis of our BMMSC.

In the context of these findings, BMMSC CM may affect various SCI-induced secondary processes such as disrupted vessel continuity, damaged blood/spinal cord barrier integrity, neuronal apoptosis, ongoing demyelination and inflammation [17,34,35]. In particular, our functional study confirmed that BMMSC CM enhances gene-encoding proangiogenic factors, such as FGF2, LIF, HGF, ICAM1, IGF1, TGFB3 and receptors (NGFR, PDGFRB) associated with increasing blood vessel density in a CAM model [25]. Restoration of oxygen and nutrient levels in damaged spinal cord tissue might be one of the mechanisms by which BMMSC CM may beneficially influence neurological outcomes in SCI dogs [36]. This is in accord with similar findings confirming the favorable action of BMMSC CM on large blood vessel growth in two experimental SCI models [13,35].

It is important to mention that extracellular vesicles (EV) and their major type exosomes, which originate from endosomal intraluminal vesicles, are an important component of the paracrine factors present in the conditioned medium derived from stem cells [37,38]. EV are small spherical structures (50–150 nm diameter) protected against degradation and digestion by a double lipid membrane [39]. Thus they can communicate with other cells, freely pass through the blood–spinal cord barrier and reach the lesion site. Their ability to deliver several active components of their cargo such as proteins, lipids, mRNAs, microRNAs and DNA into the damaged spinal cord tissue may stimulate neuroprotective or neuroregenerative processes [25,39,40]. In correlation with these findings, several experimental studies delivering BMMSC-derived EV have confirmed improved blood–spinal cord barrier (BSCB) integrity and permeability leading to better functional recovery after SCI [41,42,43].

Furthermore, BMMSC-EVs can inhibit neuronal apoptosis after administration for SCI by activating the Wnt/β-catenin signaling pathway [44] 

Because our primary goal was to assess the feasibility and partially also the efficacy and safety of BMMSC CM in SCI dogs, elucidating the precious mechanisms of action was beyond the scope of this study due to the limited number of animals included.

In the present study we; therefore, focus on developing an optimal delivery protocol for allogenic BMMSC CM by determining the optimal dose, number of administrations and time variance between individual doses. Following our preliminary studies, we decided to deliver 1–2 mL BMMSC CM/kg body weight by slow i.v. infusion in saline, which was well tolerated by all dogs without adverse effects. Since BMMSC CM is a metabolome of cells whose biological activity may be depleted after a certain time, it was necessary to repeat the administration of BMMSC CM to achieve any efficacy at all [15]. With regard to our rat studies, we decided for weekly treatment over a period of one month, which was shown to be optimal for dogs [13]. Each systemic i.v. administration was carried out with the participation of the dog owner, without complications.

It is likely that comprehensive physio rehabilitation should be simultaneously involved into cell-free regenerative medicine therapy for the standard care of most musculoskeletal and neurologic injuries. Our dogs showed thoracolumbar spinal cord damage between T11 to L2. This region is considered to be unstable and most vulnerable [45]. The main movement in this area is extension and flexion, and this has to be considered when creating any physio therapeutic plan [45,46]. For dogs enrolled in this study it was; therefore, critical to make the thoracolumbar spine clinically stable to avoid additional complications and injuries [46]. The main function of rehabilitation is to strengthen muscles and core and to facilitate passive and active movement of the limbs. 

Passive movements are a crucial part of physiotherapy. They help to prevent immobile patients from stiffening and atrophy by promoting blood flow as well as sensory and proprioceptive pathways [46]. Soft tissue massage has beneficial effect on muscle tension and reduces neuromuscular excitability, thereby triggering sensory receptors [47]. Massage relieves pain through modification of inflammatory signal pathways, nerve sensation and prevention of secondary injury [48]. Heat therapy also seems to be good pain reliever, and it can be used to supply some analgesic drugs as well [49,50]. Electro-acupuncture is considered part of passive training, and in a rat study in combination with application of MSCs it was seen to lead to neuronal regeneration, stem cell differentiation and neurofilament nerve fiber positivity in the area of SCI [51]. Electro-acupuncture also suppressed astrocyte proliferation and inhibited formation of glial scar in experimental SCI rats [52]. We used electro-acupuncture to support bladder function, which improved urination control in two dogs. Acupuncture is effective in humans with neurogenic bladder, when after three weeks of electro-acupuncture patients showed more balanced bladder function than patients without this procedure [53,54]. Functional electrical stimulation (FES) in general enhances regeneration of axons and influences better functional recovery after SCI in humans [55].

Procedures involving active movement such as hydrotherapy (under-water treadmill), balance exercises, sitting-standing position training or turns, all included in our rehabilitation plan, may improve signal transmission in nerves, stimulate muscle memory and inhibit muscle atrophy or fibrosis [46]. Hydrotherapy helps to improve core stability, muscle strength, endurance and cardiorespiratory functions [56]. Rats showed significant improvement in balance, excitability and locomotion when they underwent locomotion exercises such as “cycling” and treadmill walking after induced SCI [57]. Animals also had decreased lesion length as well as increased GABAb receptor expression and neurofilament fiber development in the area of the lesion compared to control animals [57]. The timing of rehabilitation is crucial for recovery as there are indications that early rehabilitation ameliorates motor functions and helps in rearrangement of the motor cortex [58]. Although it is generally believed that early initiation of rehabilitation is crucial for recovery after SCI, there is no doubt that physiotherapy is also beneficial for patients in the chronic stage [59,60]. Human patients with chronic SCI (one to 19 years after injury) showed better mobility and motor function in training with exoskeleton and treadmill [59]. Rehabilitation in combination with chondroitinase administration improved the perineuronal network and appeared to be effective in chronically injured rats [61]. Intensity of rehabilitation also has great impact on the recovery rate. Chronic conditions call for less aggressive, but still intensive and frequent rehabilitation [46]. Adult rats showed better dendritic structure after caudal forelimb motor cortex damage with intensive rehabilitation for five weeks [62]. Another essential part of rehabilitation is client education to achieve long-term improvement in the patient [46].

To observe the possible beneficial impact of combined therapy with BMMSC CM and rehabilitation in dogs with chronic thoracolumbar conditions, we used the Olby scoring system for quantitative evaluation of neurological outcomes [63]. The reproducibility and reliability of this scoring system has been confirmed in several studies [64]. All dogs in our study suffered paraparesis to paraplegia and were not expected to regain any improvement in gait. The neurological status of these dogs had remained unchanged for longer than six months due to intramedullary lesions confirmed with MRI, and despite additional rehabilitation and exercise. Their Olby scores were 4 to 9, and 0 in one dog with loss of deep pain sensation, but after treatment they all partially improved.

In addition, goniometric measurements generally showed better mobility in the affected pelvic limbs. This may be due to intensive rehabilitation of the dogs either in veterinary hospital or at home, which is in correlation with similar studies [65,66,67]. Hydrotherapy helps to improve range of motion (ROM), whereby injured as well as healthy dogs appeared to have wider ROM after hydrotherapy compared to what they exhibited before treadmill exercise [68]. Secondary mechanisms may also be activated by means of MSCs. Applications of these cells in humans with degenerative joint disease and dogs with osteoarthritis led to improvement in ROM in both cases [69,70]. Although our goniometric measurements indicate positive effects of applying BMMSC CM in combination with physiotherapy, there are some limitations we have to bear in mind. There are important differences between particular breeds. German Shepherds have, for instance, less motion in their tarsal joints [71], whereas border collies have a wider range of motion [72] and; therefore, it is necessary to define normal values for different breeds [72]. Although our results show beneficial impact on functional recovery, we have to consider the common impact of all included therapies, and it is impossible to identify which therapy should have higher priority over any other.

Finally, we analyzed blood chemistry and hematology parameters mainly to determine the impact of cell-free treatment combined with rehabilitation on vital organs and blood cells. We noticed changes in only two hematology parameters when comparing the results collected before and after the treatment. One dog had slightly elevated mean platelet volume (MPV) level, which returned to normal values after treatment. There can be several reasons why the MPV was elevated. There is a substantial degree of variation in platelet size in healthy animals, or it may have occurred as an artefact, or as a result of blood sample manipulation [73]. Following treatment in another dog we registered a slight decrease in mean cell volume (MCV), termed as microcytic. There are many reasons causing microcytosis, ranging from artefact through breed association and iron deficiency to various pathological conditions [74]. However, for both dogs these small changes in hematology reference parameters may be considered more likely as artefact. In total, these results of monitored blood chemistry and hematology parameters showed that conditioned medium from BMMSC did not have any harmful effect on the clinical outcome in the dogs.

## 4. Materials and Methods

### 4.1. Canine BMMSC Characteristics and Conditioned Medium (CM)

Bone marrow was isolated from healthy adult dogs (*n* = 2) after informed consent of the owners was obtained. Procedure for isolating bone marrow was the same as described in our previous study [20].

#### 4.1.1. Flow Cytometry with Canine BMMSC CD Markers

Isolated BMMSC were cultured under standard conditions at a density of 5 × 10^5^ cells/cm^2^ in Dulbecco’s modification of Eagle’s (DMEM) medium (Thermo Fisher Scientific, Waltham, Massachusetts, USA) supplemented with 10% fetal bovine serum (FBS), 100 units/mL penicillin, 100 mg/mL streptomycin (Thermo Fisher Scientific, Waltham, Massachusetts, USA) in T75 tissue culture flasks and incubated at 37 °C, and 5% CO_2_. After two weeks in culture and changing fresh medium every three days, BMMSC at passages 3 and 4 were detached using 0.025% trypsin for 5 min at 37 °C. Collected cells were identified by means to direct staining with a subsequent combination of conjugated monoclonal antibodies: anti-CD45-FITC + anti-CD29-R-PE + anti-CD90- allophycocyanin (APC), or with isotype control immunoglobulins (IgGs, 1 μg each). Samples containing 1 × 105 cells in 100 μL PBS were stained with 5 μL anti-dog CD45/IgG2b (Clone: YKIX716.13, BIOPORT, CZ), 5 µL anti-human CD29/IgG1 (Clone: TS2/16, reactivity: human, canine, Sony Biotechnology, San Jose, California, USA), and 5 μL anti-dog CD90/IgG2b (Clone: YKIX337.217, BIOPORT, CZ). For isotype controls we used FITC conjugated dog IgG (for CD29) and phycoerythrin (PE) or APC conjugated dog IgG2b (for CD45 or CD90) from Biolegend (San Diego, CA, USA). The tubes were incubated for 45 min in the dark at laboratory temperature. Then the tubes were washed twice with 1 mL PBS (MP Biomedicals, Illkirch-Graffenstaden, France) followed by centrifugation for 5 min at 250× *g*, and subsequently 100 μL of PBS was added to each tube. Flow cytometric analysis was carried out using BD FACS DivaTM analysis software on a BD FACSCanto™ flow cytometer (Becton Dickinson Biosciences, San Jose, CA, USA). The proportions of cells expressing the mentioned CD markers are expressed in percentages in histograms.

#### 4.1.2. Three-Lineage Profile of BMMSC

The multilineage capacity of canine BMMSC (passage 3) was investigated with commercial Stem Pro Differentiation Kits, which contain all the reagents required for canine BMMSC differentiation towards chondrogenic, osteogenic and adipogenic lineages. Samples were induced with the appropriate differentiation medium for each specific lineage over 14 days in accordance with the manufacturer’s instructions. Finally, the cultures were fixed with 4% formaldehyde and stained with the following reagents: Osteogenic culture with Alizarin Red S, chondrogenic culture with Alcian Blue and adipogenic culture with Oil Red (all from Sigma-Aldrich, St. Louis, MO, USA).

#### 4.1.3. BMMSC-Derived Conditioned Medium Preparation

Expanded cultures of BMMSC at passage 3 and 4 were washed with PBS in order to replace the previous cultivation medium. Confluent BMMSC (80–90%) in T75 tissue culture flasks were incubated in 5ml DMEM with low glucose and without FBS in a humidified atmosphere with 5% CO_2_ at 37 °C for 24 h and used for BMMSC conditioned medium (CM). Afterwards the supernatant was removed, centrifuged 5 min (500× *g*) and filtered through a 0.22 μm Millipore (GS) membrane. The BMMSC conditioned medium was aliquoted, and stored at −80 °C until use.

### 4.2. Dogs Suffering from SCI

All dogs enrolled in the present study had to meet our inclusion/exclusion criteria. The main inclusion criterion for our study was: chronic spinal cord injury which had not improved after standard treatment and rehabilitation. The exclusion criterion was any record of tumor or systemic disease. Based on the clients’ signed informed consents we enrolled four client-owned dogs with chronic SCI due to thoracolumbar intramedullary lesion. The dogs had undergone previous decompression spinal surgery, and the neurological status of these dogs was unchanged for at least six months before enrolment. The dogs enrolled were of different breeds (Shi-tzu, German Shepherd, Yorkshire Terrier, Bichon), 4–6 years old, both sexes, two males and two females with body weight ranging between 4–24 kg (Table 3). These dogs were selected from regular patients visiting the Veterinary University Hospital at the University of Veterinary Medicine and Pharmacy (UVMP) in Kosice, Slovakia. The animal study was reviewed and approved by the Scientific and Clinical Review Board of UVMP in Kosice, Slovakia, and involved client-owned animals only. The study was carried in accordance with the European Communities Council Directive (2010/63/EU) regarding the use of animals in research, and Slovak Law for Animal Protection 377/2012 and 436/2012. Written informed consent was obtained from the owners for the participation of their animals in this study. We adhered to a high standard of best practice veterinary care to ensure that all studied dogs received the best care available.

### 4.3. Clinical and Neurological Observation

All dogs underwent thorough clinical and neurological examination (posture and gait, cranial nerves, postural reaction, spinal reflexes, sensitivity, and spinal palpation). Ultrasound and x-ray examinations were used to exclude tumors, and hematology and biochemistry to rule out any systemic disease. All dogs were assessed as clinically healthy with intramedullary spinal cord lesion affecting their hind-limb function.

### 4.4. Collection of Blood Samples

Fresh blood samples (4 mL/dog) were collected by experienced veterinarians from the dogs (*n* = 4), from the cephalic vein and were collected in serum-separator tubes (4.4 mL, 75 × 13 mm, Z-Gel, Sarsted AG and Co KG, Nümbrecht, Germany) and in tubes containing anticoagulant (K3E 1.3 Micro tube, 1.6 mL EDTA/mL, Sarsted AG and Co KG, Nümbrecht, Germany). The serum-separator tubes were allowed to stand for 15–30 min at room temperature and afterwards were centrifuged for 10 min at 3500× *g*. Separated serum was collected and processed for chemical analyses with CATALYST ONE (IDEXX Laboratories, Inc., ME, USA). We used a basic Chem 10 Clip from Idexx (IDEXX Laboratories, Inc., Westbrook, ME, USA) which contains these basic chemical parameters: GLU, CREA, UREA, BUN/CREA, TP, ALB, GLOB, ALB/GLOB, ALT, ALP. Complete blood count (CBC) was analysed immediately after drawing fresh blood with a ProCyte Dx Hematology Analyzer (IDEXX Laboratories, Inc., ME, USA). We detected 27 whole blood parameters. Examination of blood samples was performed prior to the BMMSC CM administration and after treatment.

### 4.5. Locomotor Function

All dogs were scored with the Olby locomotor scale before the treatment and one, three and six months after the treatment. Locomotor function was evaluated using the 14-point Olby scale, where 0 indicates complete paraplegia and 14 indicates normal gait [63]. The dogs were filmed walking on a non-slippery floor in a position where the hind limbs could be clearly viewed. The final scores from the locomotor tests were obtained from three independent evaluators/dog, thus mean values were used in the final locomotor graph.

### 4.6. Imaging Methods-MRI

**Figure 5:**
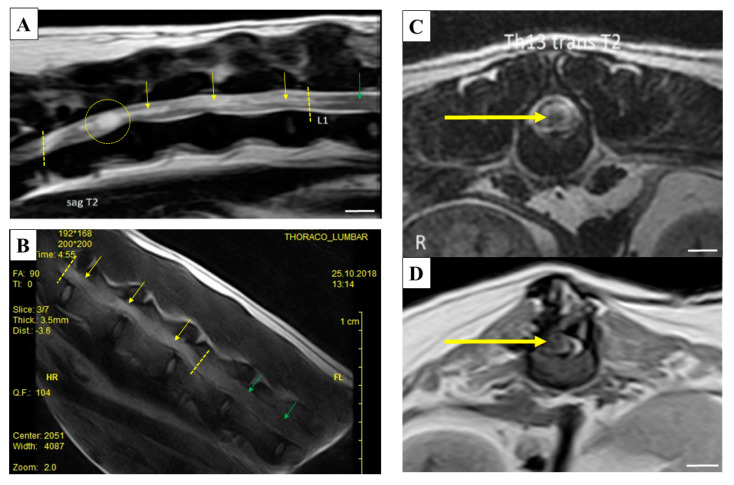
Magnetic resonance imaging (MRI) images of thoracolumbar segments of dogs with spinal cord injury (SCI). (**A**) Sagittal T2-weighted image of the thoracolumbar spine in Dog Z. Hyperintense diffused intramedullary lesion is visible from vertebra T11 to L1. At the level cranially to the T11–T12 intervertebral disc space there is a cyst-like lesion affecting the whole spinal cord diameter. (**B**) Sagittal SSE image of the thoracolumbar spine in Dog B. Hyperintense diffused intramedullary lesion is visible at the level from T13 to L2 vertebra. (**C**) Transversal T2-weighted image of Dog T spine at the level of T13 vertebra. Focal hyperintense intramedullary lesion with hypointense center is visible at the level of vertebra T13–L1 (yellow circle). (**D**) Transversal T1-weighted image of Dog M spine at the level of T12 vertebra. Focal hypointense intramedullary lesion is visible at the level of vertebra T11–T12. This lesion was also hyperintense on T2-weighted images (yellow arrows point to damaged spinal cord tissue, green arrows to normal tissue). Scale bars A, C and D = 1 cm.

Magnetic resonance imaging of the spinal cord allowed us to complete the neurological assessment. MRI was performed at a specialized clinic using 1.5 Tesla, high-field MRI equipment (GE). The dogs were positioned in dorsal recumbence. The entire vertebral column and spinal cord was examined in sagittal T2 and T1 sequences. The abnormal regions were examined in dorsal and transversal T2 FSE sequences, transversal T1 FSE and transversal T2 GRE sequences, as well as pre- and post-contrast transversal T1 FSE and post-contrast sagittal T1 FSE sequences. The common features in all four dogs were (i) that almost all intervertebral discs had lower signal intensity in T2w sequences, and (ii) that there were no significant disc protrusions or extrinsic spinal cord compression in the entire vertebral column. The spinal cord of each dog in the affected segment had a diffused or focal intraparenchymal (intramedullary) lesion with increased signal intensity in T2w sequences, but no significant spinal cord swelling. These lesions were either isointense or hypointense in T1w sequences. There was no significant contrast enhancement in the other, uninjured spinal cord regions. The affected area of the spinal cord in Dog Z was at the level of vertebral segments from T11 to L1, Dog B suffered diffused lesion at the level from T13 to L2 vertebral segments. Dog T was diagnosed with a focal lesion at the level of T1–L1 and Dog M had a lesion at the level of T11–T12. The MRI images of these lesions are shown in Figure 5A–D.

### 4.7. Treatment

**Figure 6:**
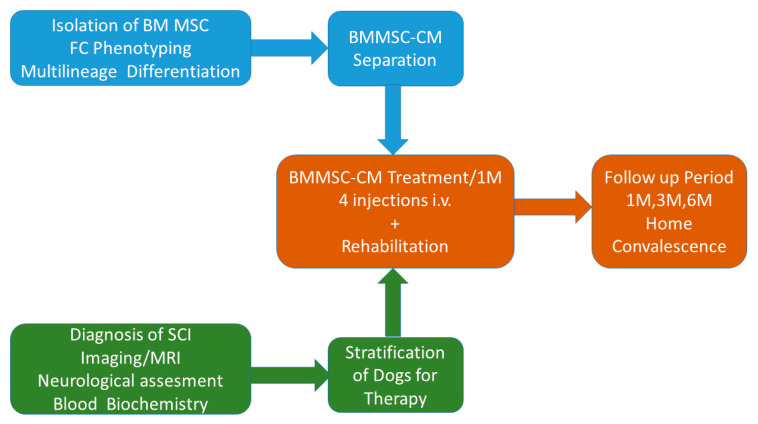
Design of treatment protocol. Chronological procedures performed before, during and after bone marrow tissue-derived MSC conditioned medium (BMMSC CM) treatment. FC (flow cytometry), M (month), i.v. (intravenous), SCI (spinal cord injury), MRI (magnetic resonance imaging).

Conditioned medium from BMMSC, concentration 1 × 10^6^ BMMSC/1 mL CM, was defrosted and diluted at dose 1–1.5 mL/kg of body weight in a total of 15–30 mL of saline (0.9% NaCl, Envibag, Imuna Pharm a.s. Sarisske Michalany, Slovakia). Infusion solution containing BMMSC CM was slowly delivered intravenously over approximately 25–40 min in awake dogs which were accompanied by their owner (infusion rate = 25–35 mL/h). During CM delivery, each dog was monitored (body temperature, blood pressure, and heart rate) and kept at our clinic under observation for an additional 30 min after the infusion for post-treatment monitoring. Treatment was repeated every week for one month, with a total of four treatments per dog. Owners of the dogs were in daily contact with a veterinarian during the entire study period. All dogs underwent the same treatment protocol (Figure 6).

### 4.8. Rehabilitation

All dogs underwent specialized physiotherapy from 24 h after BMMSC CM infusion. The physiotherapeutic plan consisted of the following modalities, which were adjusted according to the patients’ abilities during time and recovery to reduce muscle spasm, maintain soft tissue flexibility, and increase sensory awareness, strength and balance. Later the patients proceeded to exercises activating core muscles, improving balance and coordination, enhancing gait patterns, and increasing exercise tolerance and cardiovascular fitness, core stability and strength. Physiotherapeutic procedures were chosen in relation to the clinical findings and clinical state of the patients. All dogs underwent soft tissue massage and heat therapy in order to reduce muscle spasm. They underwent hydrotherapy on an underwater treadmill with commencing duration and frequency of 10 min per week and increasing duration up to 20 min depending on each patient’s response. Neuromuscular electrical stimulation was applied in three dogs except Dog B, as electrical stimulation is not recommended in patients with a history of seizures (Dog B was diagnosed with idiopathic epilepsy). Electrical stimulation was applied with an acupuncture pen (E-Acu-Pen) at acupuncture points BL26, LU7, ST25 and ST35 to help release the urinary retention. All patients underwent balance exercises on fit-ball and balance disc to increase their strength, balance and core stability. Patients proceeded first through passive exercises for joint range, and after amelioration through active exercises to maintain flexibility, coordination and strength. Physiotherapy was applied between BMMSC CM treatment cycles and continued individually for an additional three months. Owners were instructed to perform a home exercise program with passive exercises, stretches and standing training until six months after the treatment. Advice was given regarding walking with a mobility aid (e.g., harness and sling if required).

### 4.9. Goniometric Assessment

Goniometry was used to measure the range of movement before and after BMMSC CM treatment. Goniometric measurements were performed by an experienced surgeon; the patients were not sedated. We measured movement in the dogs in both lateral recumbences, always measuring the leg not lying on the mat. Maximum and minimum (extended and flexed) angles and range of movement were obtained using a standard plastic goniometer. The measurement technique with normal ranges of movement was performed according to Millis and Levin [75]. Furthermore, as reference points for goniometric measurements we used anatomical landmarks for shoulder, elbow, carpus, hip, stifle and tarsus according to previous studies [76,77].

### 4.10. Urination Assessment

Assessment of urinary function was done subjectively based on each owner’s checklist and on the ease of manual expression and the presence of spontaneous urination. 

We created a numerical scale for the purposes of this study.

Score of urinary function: (used for Dog Z and Dog M)

1. Complete loss of urinary function, manual expression needed at least three times daily, and it is hard to express the bladder manually;
2. Partial recovery of urinary function, partial release of sphincter spasm, easier to express;
3. Marked recovery of urinary function, release of sphincter spasm, easy to express, dog stretches tail and flexes legs when the bladder is manually expressed;
4. Pronounced recovery of urinary function, marked release of sphincter spasm, very easy to express, dog sometimes urinates after irritation (tickle) of the perineum and seems nervous when the bladder is full;
5. Complete recovery of urinary function, physiological urination outside the house, dog is always nervous with a full bladder.

Two dogs (Dog Z and Dog M) were unable to urinate spontaneously prior to treatment; they suffered from urinary retention due to the existing upper motor lesion. They had to be manually expressed at least three times daily; the other two dogs (Dog B and Dog T) urinated normally.

### 4.11. Statistical Analysis

Data are presented as mean ± standard error of mean (SEM); statistical significance was analyzed using SigmaPlot v. 13 software (Systat Software Inc., San Jose, CA, USA).

## 5. Conclusions

Despite enormous efforts by researchers and clinicians, no effective regenerative treatment has yet been found to treat SCI. Although MSCs have paracrine action and release a variety of growth factors and cytokines, and their anti-inflammatory and immunomodulatory actions have been shown to improve SCI in small laboratory animals, their limited regenerative capacity after SCI in dogs is more similar to the situation in humans. In the present pilot feasibility study we demonstrate that four intravenous injections of BMMSC conditioned medium, combined with subsequent rehabilitation, improved chronic SCI in dogs. Moreover, we found that application of BMMSC conditioned medium in dogs was safe and well tolerated. Rehabilitation supported by application of stem-cell conditioned medium resulted in improved hind-limb movement and bladder control. We suggest that non-invasive, multiple injections of allogenic stem-cell conditioned medium can replace cell therapy and can contribute to spinal cord regeneration, and this therapy should; therefore, be further studied for treatment of SCI. However, the safety and efficacy of the treatment need to be assessed in a long-term study involving a larger number of animals and placebo controls.

## Figures and Tables

**Table 1:** Goniometric measurements of mobility. (**A**, **B**, **C**) Measurements of flexion and extension according to the anatomical coordinates for shoulder, elbow, carpus, hip, stifle were performed in Dogs Z, B and M successively.

A						
Dog Z		Right Limb		Left Limb		Normal Ranges
		Before Treatment	After Treatment	Before Treatment	After Treatment	
Shoulder	*flexion*	30 ± 1	40 ± 1.7	25 ± 1.7	30 ± 1.7	30–60
	*extension*	140 ± 1.7	175 ± 2	165 ± 1	176 ± 1.7	160–170
Elbow	*flexion*	35 ± 1.7	20 ± 1.7	30 ± 1	20 ± 2	20–40
	*extension*	175 ± 1	155 ± 1	160 ± 1.7	170 ± 3.4	160–170
Carpus	*flexion*	16 ± 1	15 ± 1	16 ± 1	14 ± 1	20–35
	*extension*	220 ± 3.4	185 ± 1.7	220 ± 2.6	190 ± 1	190–200
Hip	*flexion*	39 ± 2.6	39 ± 1	40 ± 1.7	40 ± 1	55
	*extension*	174 ± 2	140 ± 1.7	170 ± 2	157 ± 1	160–165
Stifle	*flexion*	20 ± 1	10 ± 1	25 ± 1.7	17 ± 1.7	45
	*extension*	170 ± 1	175 ± 1	182 ± 1	168 ± 1	160–170
Tarsus	*flexion*	25 ± 1	25 ± 1	15 ± 1	15 ± 1	40
	*extension*	165 ± 1	190 ± 1	185 ± 1.7	185 ± 1	170
**B**						
**Dog B**		**Right Limb**		**Left Limb**		**Normal Ranges**
		**Before Treatment**	**After Treatment**	**Before Treatment**	**After Treatment**	
Shoulder	*flexion*	48 ± 1	55 ± 2.6	89 ± 1.7	45 ± 2	30–60
	*extension*	137.5 ± 0.8	160 ± 2.6	178 ± 2	169 ± 1.3	160–170
Elbow	*flexion*	26 ± 1	20 ± 2.6	25 ± 2	19 ± 1.7	20–40
	*extension*	143 ± 1	162 ± 1.5	175 ± 1.7	163 ± 1.7	160–170
Carpus	*flexion*	14 ± 1.7	16 ± 2	15 ± 1.7	16 ± 1	20–35
	*extension*	163 ± 1	190 ± 2	175 ± 2	180 ± 2	190–200
Hip	*flexion*	10 ± 2	10 ± 1.7	38 ± 1	32 ± 1	55
	*extension*	145 ± 1	129 ± 2	96 ± 2	145 ± 2.6	160–165
Stifle	*flexion*	22 ± 2.6	25 ± 2	40 ± 2	45 ± 1.7	45
	*extension*	130 ± 1.7	145 ± 1.7	135 ± 1.7	155 ± 3	160–170
Tarsus	*flexion*	30 ± 2	39 ± 2	20 ± 2.6	28 ± 1	40
	*extension*	145 ± 1.7	153 ± 2	145 ± 1	168 ± 1.7	170
**C**						
**Dog M**		**Right Limb**		**Left Limb**		**Normal Ranges**
		**Before Treatment**	**After Treatment**	**Before Treatment**	**After Treatment**	
Shoulder	*flexion*	45 ± 1	35 ± 2	49 ± 1.7	35 ± 2	30–60
	*extension*	185 ± 2	140 ± 1.7	200 ± 2	170 ± 2.6	160–170
Elbow	*flexion*	35 ± 2	30 ± 1.7	28 ± 1	30 ± 1	20–40
	*extension*	218 ± 1.7	184 ± 1.7	183 ± 2	210 ± 2	160–170
Carpus	*flexion*	19 ± 2	20 ± 2	19 ± 2	19 ± 1	20–35
	*extension*	168 ± 2	124 ± 1.7	210 ± 2	235 ± 2	190–200
Hip	*flexion*	13 ± 1	10 ± 1	8 ± 1	20 ± 2	55
	*extension*	187 ± 1	180 ± 1.7	169 ± 1	180 ± 2.6	160–165
Stifle	*flexion*	19 ± 2	22 ± 1	10 ± 1.7	20 ± 1	45
	*extension*	184 ± 1	170 ± 2	175 ± 2.6	130 ± 2	160–170
Tarsus	*flexion*	35 ± 1.7	40 ± 1.7	25 ± 1.7	30 ± 1.7	40
	*extension*	185 ± 1	185 ± 1.7	173 ± 2	190 ± 1.7	170

**Table 2:** Hematology parameters before treatment (BT) and after treatment (AT), RBC (red blood cells), HCT (hematocrit), HGB (hemoglobin), MCV (mean corpuscular volume), MCH (mean corpuscular hemoglobin), MCHC (mean corpuscular hemoglobin concentration), RDW (red cell distribution width), RETIC (reticulocytes), WBC (white blood cells), NEU (neutrophils), LYM (lymphocytes), MONO (monocytes), EOS (eosinophils), BASO (basophiles), PLT (platelets), MPV (mean platelet volume), PDW (platelet distribution width), PCT (plateletcrit).

Hematological Parameters	Dog B		Dog M		Dog T		Dog Z		Normal Ranges
	BT	AT	BT	AT	BT	AT	BT	AT	
RBC (×10^12^/L)	5.97	8.09	7.53	7.97	8.75	6.27	6.24	7.35	5.65–8.87
HCT (%)	39.1	51.3	48.4	51.3	55.6	38.1	39.3	50.7	37.3–61.7
HGB (g/DL)	14.1	17.6	16.2	17.3	19	134	13.2	16.6	13.1–20.5
MCV (fL)	65.5	63.4	64.3	64.4	63.5	60.8	63	69	61.6–73.5
MCH (pg)	23.6	21.8	21.5	21.7	21.7	21.4	21.2	22.6	21.2–25.9
MCHC (g/DL)	36.1	34.3	33.5	33.7	34.2	35.2	33.6	32.7	32–37.9
RDW (%)	14.7	18.9	18.3	18.1	18.9	15.9	14.4	14.9	13.6–21.7
%RETIC	0.8	0.7	0.7	0.9	0.1	0.2	0.3	0.5	
RETIC (K/µL)	44.8	57.4	48.9	73.3	12.3	15	20.6	33.8	10–110
RETIC-HGB (pg)	27.6	25.9	24.4	22.4	25.6	23.6	241	25.5	22.3–29.6
WBC (×10⁹/L)	9.91	11.1	9.7	9.18	7.52	15.46	11.93	6.22	5.05–16.76
%NEU	67.7	74.7	71.1	71	72	72.2	75.5	65	60–77
%LYM	20.6	16.6	21.2	23.3	21.9	19.1	11.7	26.8	12–30
%MONO	6.6	4.4	6.8	3.5	3.3	5.9	4.4	5.5	3–10
%EOS	5.1	3.9	0.8	2.1	2.7	2.7	8.2	1.1	2–10
%BASO	0	0.4	0.1	0.1	0.1	0.1	0.2	1.6	0–1
NEU (×10⁹/L)	6.71	8.3	6.89	6.52	5.41	11.16	9.01	4.04	2.95–11.64
LYM (×10⁹/L)	2.04	1.84	2.06	2.14	1.65	2.95	1.39	1.67	1.05–5.10
MONO (×10⁹/L)	0.65	0.49	0.66	0.32	0.25	0.91	0.53	0.34	0.16–1.12
EOS (×10⁹/L)	0.1	0.43	0.08	0.19	0.2	0.42	0.98	0.07	0.06–1.23
BASO (×10⁹/L)	0	0.04	0.01	0.01	0.01	0.02	0.02	0.1	0.00–0.10
PLT (K/µL)	376	254	164	348	245	293	387	268	148–484
MPV (fL)	12	11.6	16.7	10	11.8	11.8	11.2	10.2	8.7–13.2
PDW (fL)	11.9	10	not measured	10.2	11.3	10.5	9.4	10.7	9.1–19.4
PCT (%)	0.45	0.9	0.27	0.35	0.29	0.35	0.43	0.3	0.14–0.46

**Table 3:** An overview of dogs enrolled in the study.

Breed	Dogs Name	Lesion Site	Age	Gender	Body Weight
Shi-tzu	Zuzi (Dog Z)	T11-L1	4	Female	5.4 kg
German Shepherd	Bak (Dog B)	T13-L2	6	Male	24 kg
Yorkshire Terrier	Max (Dog M)	T11-T12	5	Male	4 kg
Bichon	Tia (Dog T)	T13-L1	4	Female	6.4 kg

## Abbreviations

ALB	Albumine
ALB/GLOB	Ratio albumine/globuline
ALKP	Alkaline phosphatase
ALT	Alanine aminotransferase
BMMSC	Bone marrow tissue-derived MSC
BMMSC CM	Bone marrow tissue-derived MSC conditioned medium
BDNF	Brain-derived growth factor
BUN/CREA	Ratio urea/creatinine
CBC	Complete blood count
CNS	Central nervous system
CM	Conditioned medium
CREA	Creatinine
CSF	Cerebrospinal fluid
DMEM	Dulbecco’s modification of Eagle’s medium
EDTA	Ethylene diamine tetra acetic acid
EV	Extracellular vesicles
FBS	Fetal bovine serum
GLOB	Globuline
GLU	Glucose
IVDD	Intervertebral disk
MRI	Magnetic resonance imaging
MPV	Mean platelet volume
MSC	Mesenchymal stromal cells
PBS	Phosphate-buffered saline
SCI	Spinal cord injury
TP	Total protein
UREA	Urea
